# Cardiovascular complications and outcomes among athletes with COVID-19 disease: a systematic review

**DOI:** 10.1186/s13102-022-00464-8

**Published:** 2022-04-20

**Authors:** Bandar Alosaimi, Isamme AlFayyad, Salman Alshuaibi, Ghazwaa Almutairi, Nawaf Alshaebi, Abdulaziz Alayyaf, Wael Alturaiki, Muhammad Azam Shah

**Affiliations:** 1 Research Center, King Fahad Medical City, Riyadh, 11525 Saudi Arabia; 2 College of Medicine, Imam Mohammad Ibn Saud Islamic University, Riyadh, 13317 Saudi Arabia; 3 College of pharmacy, Qassim University, Qassim, 51911 Saudi Arabia; 4 Faculty of Medicine, King AbdulAziz University, Jeddah, 21589 Saudi Arabia; 5 Faculty of Medicine, Prince Sattam bin Abdulaziz University, Alkharj, Riyadh, 11942 Saudi Arabia; 6 Department of Medical Laboratory Sciences, College of Applied Medical Sciences, Majmaah University, Majmaah, 11952 Saudi Arabia; 7 Adult Cardiology Department, King Salman Heart Center, King Fahad Medical City, Riyadh, 11525 Saudi Arabia; 8 Research Center, King Fahad Medical City, Riyadh, 11525 Saudi Arabia

**Keywords:** Cardiovascular, COVID-19, Athletes, Rehabilitation, Extrapulmonary

## Abstract

**Background:**

Current evidence still emerging regarding the risk of cardiovascular (CV) sequel associated with coronavirus disease 2019 (COVID-19) infection, and considerable replicated studies are needed to ensure safe return-to-play. Therefore, we aimed in this systematic review to measure the prevalence of CV complications suffered by COVID-19 athletic patients, explore the outcomes, optimal approaches to diagnoses, and safe return-to-play considerations.

**Methods:**

A systematic search on post COVID-19 infection quantitative studies among athletes was conducted following MeSH terms in Medline, Cochrane Library, Ovid, Embase and Scopus (through 15 January 2022). We included peer-reviewed studies reported athletes’ CV complications and the outcomes post COVID-19 infection. Editorials, letters, commentaries, and clinical guidelines, as well as duplicate studies were excluded. Studies involving non-athletic patients were also excluded. Quality assessment was performed using Newcastle–Ottawa Scale.

**Results:**

We included 15 eligible articles with a total of 6229 athletes, of whom 1023 were elite or professional athletes. The prevalence of myocarditis ranged between 0.4% and 15.4%, pericarditis 0.06% and 2.2%, and pericardial effusion between 0.27% and 58%. Five studies reported elevated troponin levels (0.9-6.9%).

**Conclusions:**

This study provides a low prevalence of CV complications secondary to COVID-19 infection in short-term follow-up. Early recognition and continuous assessment of cardiac abnormality in competitive athletes are imperative to prevent cardiac complications. Establishing a stepwise evaluation approach is critical with an emphasis on imaging techniques for proper diagnosis and risk assessment for a safe return to play.

## Background

Severe acute respiratory syndrome coronavirus 2 (SARS-CoV-2) has affected nearly 372,875,793 million people (as of 30 January 2022) worldwide since its outbreak in China in December 2019 [1]. COVID-19 affects the respiratory system and infected people usually experience a relatively mild course of symptoms such as fever, headache, cough, shortness of breath, and diarrhea [2]. However, severe infection manifests to the point of causing severe pneumonia and often progresses to acute respiratory distress syndrome (ARDS) [3].

COVID-19 is considered to be a multi-organ disease that could lead to a broad variety of clinical complications affecting multiple body systems, such as the cardiovascular (CV) system [4]. COVID-19 is associated with a range of CV complications, specific arrhythmias, myocardial injury, and other cardiovascular diseases, with potentially fatal outcomes in athletes and non- athletes [4, 5].

A study by Saurabh Rajpal and colleagues in 2020 used cardiac magnetic resonance to visualize the cardiac complications in 26 athletes who have recovered from COVID-19 infection. Among 26 athletes, 4 had cardiovascular magnetic resonance (CMR) imaging outcomes that were indicative of myocarditis, and 8 athletes displayed late gadolinium enhancement (LGE) without T2 elevation, indicative of pre-myocardial injury [6]. Emerging knowledge and cardiac imaging observations raised concerns of myocardial inflammation as an additional cause of cardiac damage from COVID-19. Therefore, practical recommendations have proposed a medical assessment tool that investigate the cardiorespiratory complications and the severity of illness suggesting a roadmap to exclude cardiorespiratory complications of COVID-19 in athlete [7]. Moreover, animal experiments have demonstrated that exercise can increase virus replication and inflammation inside the heart that is affected by myocarditis, resulting in irreversible injury or occasionally sudden death [7]. In addition to that, even within asymptomatic or mildly symptomatic patients, recent reports have raised questions about myocardial inflammation after recovery from COVID-19 [6]. However, the occurrence of myocarditis-induced arrhythmias is not known, and although COVID-19 has contributed to a rise in over 50% in the general population of hospital cardiac arrests, the data do not indicate a rise in the risk of sudden cardiac arrest or arrhythmias in otherwise healthy COVID-19 patients [7]. Even asymptomatic or relatively mild symptomatic COVID-19 patients show cardiac magnetic findings consistent with myocarditis myocardial inflammation [8]. The cardiac involvement in athletics with SARS-CoV-2 such as myocarditis manifests histologically with lymphocytic infiltrates, acute impairment of heart function, possibly residual chronic scarring with increased susceptibility to malignant ventricular arrhythmias and other cardiovascular diseases [4, 5]. We aimed to accumulate the available evidence concerning the post-recovery cardiac complications suffered by COVID-19 athletic patients.

## Methods

### Search strategy

**Fig. 1:**
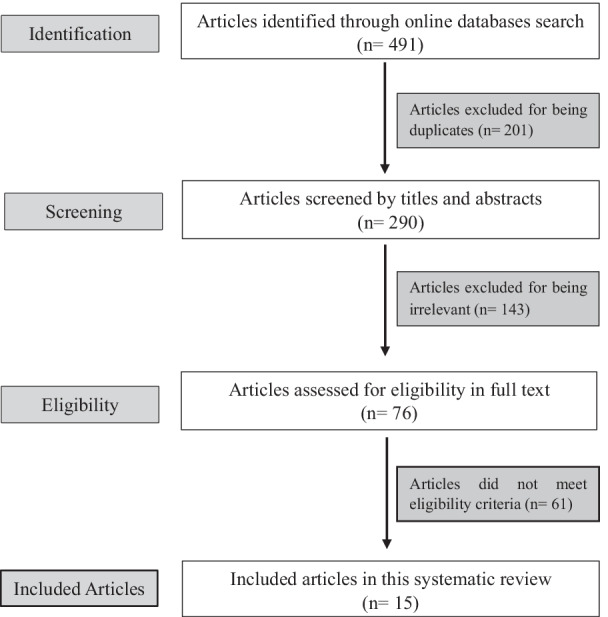
PRISMA flow chart describing selection of studies included in the systematic review

We conducted a systematic literature search of online databases including in Medline, Cochrane Library, Ovid, Embase and Scopus for articles published between January 1st 2020 to January 28, 2022. Following Preferred Reporting Items for Systematic Reviews and Meta-analysis (PRISMA) guidelines (Fig. 1), we identified all articles that discussed post-recovery cardiac complications in COVID‐19 infected athletes. We searched for the following keywords as MeSH terms: “COVID‐19”, “coronavirus”, OR “SARS-CoV-2” in combination with terms “Myocarditis”, “Pericarditis”, “myopericarditis”, “heart failure”, “exercise”, “athletes”, “sport”, “post-recovery”, and “complications”.

A total of 491 records were identified through our systematic search. Duplicate results were removed. The remaining articles (n = 201) were screened for relevance by their title and abstracts by four authors (G.A, S.A, N.A and A.A). Disagreement during the inclusion and exclusion process, data extraction, and quality assessment was resolved by consensus or involvement of a fifth author (B.A). Seventy-six articles were assessed in full text for eligibility, of which 15 articles were included in this systematic review and identified as relevant.

### Inclusion and exclusion criteria

After identifying 491 relevant articles by keywords through online databases search, 201 articles were removed because of duplication in different databases. Screening 290 articles by titles and abstracts reveled an exclusion of 143 articles for irrelevance to the scope of the review. Seventy-six articles were assessed in full text for eligibility and 61 articles were removed for one of the following reasons (1) studies using non original data e.g. editorials, narrative letters, narrative commentaries, and clinical guidelines; (2) studies that are not peer-reviewed; (3) studies involving non-athletic patients; (4) studies not reporting the CV complications and outcomes; or (5) studies not exploring approaches to diagnoses, and safe return-to-play considerations.

### Quality assessment

The quality assessment of included studies was performed using the Newcastle–Ottawa Scale (NOS) for cohort and cross-sectional studies. The NOS is a reliable system used to measure biases in quantitative studies by using its star rating system.

Selected cohort studies were evaluated for the selection of study groups (0–4 stars), comparability for confounding factors (0–2 stars), and outcome of interest ascertainment (0–3 stars), with a maximum of 9 stars representing the highest methodological quality. Each of the included cross-sectional studies was evaluated for the selection of study sample (0–5 stars), comparability for confounding factors (0–2 stars), and outcome of interest ascertainment (0–3 stars), with a maximum of 10 stars indicating the highest methodological quality. Two authors independently conducted the quality assessment for each included study. Disagreements, if any, were discussed and resolved by the involvement of a third author to reach a final judgment.

### Data extraction

Data extraction was conducted by two authors (I.F and B.A), we used standardized forms that include author, year, participants number, participants mean age, study setting, study design, COVID-19 status (intensity). The outcomes of interest were the diagnostic procedures and findings, CV complications, and recovery status among athletes with COVID-19 infection.

## Review of relevant literature

### Search results

Included studies comprised of 8 cross-sectional studies [6, 9–15], 6 cohort studies (5 studies were retrospective) [8, 16–20], and one case series study [21]. Amongst these 15 articles, nine were reported from the United States [6, 8–11, 13, 15, 19, 21], four articles from Italy [14, 17, 18, 20], one from Hungary [12], and one from Poland [16]. Seven articles were reported from elite or professional athletes [12, 14–17, 19, 20] and 8 from college athletes [6, 8–11, 13, 19, 21]. All the studies included are summarized in Table 1.

**Table 1:** Characteristics of included studies

No	Study	Country	Study design	No. of participants	Type of athlete	Age (Mean ± SD)	Study Period*	NOS Quality Score
1	Rajpal et al. [6]	United States	Cross-sectional	26	College student athletes	19.5 ± 1.5	June-August 2020	6 ¥
2	Brito et al. [9]	United States	Cross-sectional	54	College student athletes	19	July 2020	7 ¥
3	Erickson et al. [10]	United States	Cross-sectional	170	College student athletes	19.56 ± 1.51 for Men (N = 91); 19.44 ± 1.19 for Women (N = 79)	August 1-December 30, 2020	7 ¥
4	Hendrickson et al. [11]	United States	Cross-sectional	137	Collegiate athletes	20 (18–27)	July 9-October 21, 2020	5 ¥
5	Vago et al. [12]	Hungary	Cross-sectional	12	Elite athletes	23 (20–23)	NR	4 ¥
6	Daniels et al. [13]	United States	Cross-sectional	1597	College athletes	22 (10–77)	March 1-December 15, 2020	6 ¥
7	Cavigli t al. [14]	Italy	Cross-sectional	90	Professional athletes	24 ± 10	NR	5 ¥
8	Martinez et al. [15]	United States	Cross-sectional	789	Professional athletes	25 (19–41)	May and October 2020	5 ¥
9	Małek et al. [16]	Poland	Retrospective cohort	26	Elite athletes	24	August and October 2020	6 §
10	Cavarretta et al. [17]	Italy	Cohort	30	Professional soccer players	22	2020	4 §
11	Mascia et al. [18]	Italy	Retrospective cohort	58	Professional soccer players	NA	2020	4 §
12	Clark et al. [8]	United States	Retrospective cohort	59	Collegiate athletes	20 (19–34)	NR	7 §
13	Moulson et al. [19]	United States	Prospective cohort	3018	Colleges and universities athletes	20 (2)	September 1-December 31, 2020	7 §
14	Gervasi et al. [20]	Italy	Retrospective cohort	18	Professional soccer players	22 (21–27)	NR	6 §
15	Starekova et al. [21]	United States	Case series	145	College student athletes	19.6 ± 1.3	January 1- November 29, 2020	-

*Study period represents the period when the participants were diagnosed with COVID-19

¥ NOS for Cross-sectional studies

§ NOS for Cohort studies

Case series cannot be assessed by NOS

NR: Not reported

### Quality assessment

The study quality ratings from the modified Newcastle-Ottawa Scale are presented in Table 1. Of the 8 cross-sectional studies included in this review, 2 studies were deemed ‘good’ studies with a score of seven points [9, 10] and 5 study as satisfactory with a score of six points [6, 11, 13–15]. Two cohort studies were deemed ‘fair’ studies with a score of four points [17, 18], whereas the remaining 4 studies were regarded as ‘good’ [8, 16, 19, 20]. The quality issues identified in the ‘fair’ and ‘poor’ studies were due to lack of comparability items of control or inadequate comparators and absence of follow-up [17, 18]. In addition, the quality assessment for the case series study [21] was made “good” based on the overall judgement about the methodological quality tool suggested by Murad and colleagues [22].

### Characteristics of the included studies

All of the included studies were published in 2021 and evaluated 6229 athletes infected with COVID-19 with sample sizes ranging from 12 to 3018. The mean ages of included subjects ranged from 19 to 25 years, and one study did not report participants’ age [18]. In all studies, no mortality data were reported.

### COVID-19 diagnosis confirmation

Eight studies reported the use of Reverse transcriptase-polymerase chain reaction (PCR) for COVID-19 diagnosis [6, 9, 11, 16–18, 20, 21], another three studies used real-time PCR [12, 13, 15, 19], and three study did not indicate the method used for SARS-CoV-2 detection [8, 10, 14]. In addition to the PCR tests, five studies have tested serum IgG and IgM immune markers [9, 15, 17, 19, 20].

### COVID-19 phase (assessment phase)

Thirteen studies were conducted after the recovery from COVID-19 infection [9, 10, 12–21], and 2 studies were conducted while the participants were infected [6.11].

## Cardiac assessment

To assess CV complications, cardiac magnetic resonance imaging (MRI) was used in 14 studies [6, 8–16, 1–21], ECG in all studies [6, 8–21], echocardiography in eleven studies [6, 8–15, 17, 18, 20], cardiac enzyme (Troponin I or T) in fourteen studies [6, 8–16, 18–21], and Holter monitoring in five studies [10, 14, 17, 18, 20].

### COVID-19 symptoms severity

The definition of severity was consistent among all included papers. Thirteen studies have reported symptoms severity [6, 8–12, 14–17, 19–21]. Out of these thirteen studies, two studies included patients with severe symptoms [16, 21] and four studies with moderate symptoms [9–12, 16, 19, 21]. Two study did not report symptom severity [13, 18].

### Cardiovascular complications

Twelve studies have reported varied CV complications [6, 8–11, 13–16, 18, 19, 21]. Of which, six studies reported myocarditis with a prevalence range between 0.4% and 15.4% [6, 8, 13–15, 21], five study reported pericarditis [8, 10, 13–15] with a prevalence range between 0.06% and 2.2%. The prevalence of pericardial effusion was reported by nine studies and ranged between (0.27-58%) [6, 9, 11, 13–16, 18, 19], and Five studies reported elevated troponin levels (0.9-6.9%) [9, 11, 18, 19, 21]. The two studies conducted in Italy and Hungary among professional and elite players reported no CV complications were experienced among the players [12, 20].

**Table 2:** Summary of included studies and associated cardiac injury

Study	COVID-19 Confirmation	Time to assessment (CMR/Echo) (days)	Assessment phase ¥	Cardiac assessment	COVID-19 symptoms severity	CV Complications
Rajpal et al. [6] (n = 26)	RT-PCR	(11–53) to CMR	Ongoing symptomatic COVID-19	Troponin I, CMR, Echo, TTE, ECG	Asymptomatic: 14 (53.8%) Mild symptoms: 12 (46.2%)	Myocarditis: 4 (15.4%) Pericardial effusion: 2 (7.7%)
Brito et al. [9] (n = 54)	Immunoglobulin G (IgG) antibody and RT-PCR	27 (22–33) to CMR	Post COVID-19	Troponin-I, B-type natriuretic peptide, ESR, CRP CMR, ECG, Echocardiograph (Echo)	Asymptomatic: 16 (30%) Mild symptoms: 36 (66%) Moderate symptoms 2: (4%)	Pericardial effusion: 31 (58%) Elevated Troponin: 1 (3%)
Erickson et al. [10] (n = 170)	Not reported	Not reported	Post COVID-19	ECG, Echo*, Volume of oxygen consumption test (VO2) *, Holter ECG *, Troponin *, CMR*, Chest computed tomography (CT) *	Asymptomatic: 22 (12.9%) Mild symptoms: 116 (68.2%) Moderate symptoms: 31 (18.2%) Data missing: 1 (0.5%)	Effusive pericarditis: 2 (1.2%)
Hendrickson et al. [11] (n = 137)	RT-PCR	16 (12–34) to CMR	Ongoing symptomatic COVID-19	Troponin I, ECG, TTE, CMR	Mild symptoms: 75 (67%) Moderate symptoms: 37 (33%)	Pericardial effusion: 4 (2.9%) Elevated Troponin: 4 (2.9%) Coronary artery ectasia: 2 (1.5%)
Vago et al. [12] (n = 12)	PCR	17 (17–19) to CMR	Post COVID-19	CRP, N-terminal pro–B-type natriuretic protein, conventional cardiac troponin I (cTn), MR	Asymptomatic: 2 (16.7%) Mild/moderate symptoms: 10 (83.3%)	No CV symptoms
Daniels et al. [13] (n = 1597)	PCR	22 (10–77) to CMR 15 (11–25) to Echo	Post COVID-19	Troponin level, ECG, Echo, CMR	Not reported	Myocarditis: 37 (2.3%) Pericardial effusion: 1 (0.06%) Pericarditis: 1 (0.06%)
Cavigli et al. [14] (n = 90)	Not reported	Not reported	Post COVID-19	CBC, creatinine, ALT, AST, GGT, CPK, high-sensitivity troponin I, CRP, LDH, protein electrophoresis, D-dimer, ferritin, urine examination ECG, 24-h ambulatory ECG, Echo, Cardiopulmonary exercise testing, Chest CT, CMR	Asymptomatic: 21 (23.3%) Mild symptoms: 69 (76.7%)	Myocarditis: 1 (1.1%) Pericarditis: 2 (2.2%) Pericardial effusion: 3 (3.3%)
Martinez et al. [15] (n = 789)	Antibody/ PCR	19 (3–156) to Echo	Post COVID-19	Troponin level, ECG, TTE, CMR, Echo	Asymptomatic\ paucisymptomatic: 329 (41.7%) Not specified: 460 (58.3%)	Myocarditis: 3 (0.4%) Pericarditis: 2 (0.3%) Pericardial effusion: 3 (0.4%)
Małek et al. [16] **(n = 26)**	RT-PCR	32 (22–62) to CMR and Echo	Post COVID-19	Complete blood count (CBC), CRP, and troponin T CMR, ECG	Asymptomatic: 6 (23%) Mild symptoms: 14 (54%) Moderate symptoms: 5 (19%) Severe symptoms: 1 (4%)	Pericardial effusion: 2 (8%) Myocardial edema: 4 (15.4%) Non-ischemic late gadolinium enhancement (LGE): 1 (3.8%)
Cavarretta et al. [17] (n = 30)	IgG, IgM, RT-PCR	Not reported	Post COVID-19	Blood test anomalies, Respiratory parameters (spirometry), ECG (resting and stress-test), Echo Holter ECG, Chest CT	Asymptomatic: 30 (100%)	Not reported
Mascia et al. [18] (n = 58)	RT-PCR	(27–41) to CMR	Post COVID-19	Architect stat High Sensitive Troponin CBC, alanine transaminase, aspartate transaminase, (AST), gamma-glutamyl-transferase (GGT), creatine kinase (CPK), CPK myocardial band (CPK-MB), lactate dehydrogenase (LDH), partial thromboplastin time (PTT), international normalized ratio (INR), serum protein electrophoresis, ferritin, interleukin-6, CRP, D-dimer and urine test. ECG, Echo, Cardiopulmonary exercise test Holter ECG, CMR	Not reported	Pericardial effusion (3 mm): 1 (1.7%) Elevated Troponin: 4 (6.9%)
Clark et al. [8] (n = 59)	Not reported	21.5 (13–37) to CMR	Post COVID-19	Troponin I, ECG, Echo with strain, Contrasted CMR	Asymptomatic: 13 (22%) Mild symptoms: 46 (78%)	Myocarditis: 2 (3.4%) Pericarditis: 1 (1.7%)
Moulson et al. [19] (n = 3018)	Laboratory testing (PCR, antigen, or antibody)	33 (18–63) to CMR	Post COVID-19	ECG, Cardiac troponin assay, TTE, CMR	Asymptomatic: 887 (33%) Mild symptoms: 789 (29%) Moderate symptoms: 663 (25%) Cardiopulmonary: 337 (13%)	Elevated troponin: 24 (0.9%) Pericardial effusion: 6 (0.27%)
Gervasi et al. [20] (n = 18)	IgG, IgM, RT-PCR	15 to Echo	Post COVID-19	Complete blood count, ALT /AST, GGT, LDH, CPK, CRP, D-dimer, high-sensitivity troponin I (TnI), interleukin (IL)-6, PT, PTT, INR and creatinine Spirometry, ECG, Echo, Holter ECG, Chest CT, CMR	Asymptomatic: 6 (33.3%) Mild symptoms: 12 (66.7%)	No CV complications
Starekova et al. [21] (n = 145)	Reverse transcriptase –polymerase chain reaction (RT-PCR)	15 (11–194) to CMR	Post COVID-19	Cardiac magnetic resonance (CMR) Troponin-I, B-type natriuretic peptide, Erythrocyte sedimentation rate (ESR), C-reactive protein (CRP) Transthoracic echocardiography (TTE) Electrocardiogram (ECG)	Asymptomatic: 24 (16.6%) Mild symptoms: 71 (49.0%) Moderate symptoms: 40 (27.6%) Severe symptoms: 7 (4.8%) not documented: 3 (2.1%)	Myocarditis: 2 (1.4%) Elevated Troponin: 4 (2.8%)

**¥** According to the classification of the National Institute for Health and Care Excellence, Scottish Intercollegiate Guidelines Network, and Royal College of General Practitioners

*Tests done based on the Abnormal ECG Findings or Symptom Severity

## Discussion

Varied cardiovascular complications were reported in all retrieved manuscripts including; myocarditis, pericardial effusion, effusive viral pericarditis, and myocardial edema. In hospitalized non-athlete patients, COVID-19 has been associated with myocarditis, myocardial fibrosis, pericarditis, and edema in 19.7% of patients, indicative of poor prognosis and a risk factor of in-hospital mortality [23]. However, Cardiovascular abnormalities have been found to be persistent, even after recovery, in 78% of COVID-19 symptomatic or asymptomatic athletes detected by standardized CMR [24]. The most common abnormality was myocarditis followed by regional scar and pericardial enhancement [24]. Another study by Saurabh Rajpal and colleagues demonstrated cardiac magnetic resonance (CMR) results in 26 athletes found CMR evidence of myocarditis in 4 COVID-19 survived patients (15%), and 8 athletes displayed LGE (30%) without T2 elevation indicative of pre-myocardial injury [6]. Indeed, a recent study indicated 2 out of 22 (9%) competitive athletes with COVID-19 suffered from myocardial inflammation or fibrosis after the course of disease [8]. These findings are of major concern since myocarditis in athletes is a major cause of sport-related SCD and can happen with a normal ventricular output [6]. Therefore, it is crucial to identify these abnormalities early in the course of the disease and to appropriately treat them [25].

Early recognition and continuous assessment of cardiac abnormality in competitive athletes are imperative to prevent cardiac complications. Athletes infected with COVID-19 may experience a range of symptoms and varied disease severity (Table 2), however, short-term illness may include sore throat, myalgia, shortness of breath, fever, while some were asymptomatic [6]. Post-recovery symptoms may include cough, tachycardia, severe fatigue, ventricular arrhythmias, and depression [7]. Following the athletes’ initial clinical evaluation, additional assessments may include specialized blood panel, resting electrocardiogram (ECG), 24-hour ECG, echocardiogram, cardiopulmonary exercise, and CMR imaging interpreted by a cardiology consultant [5]. Cardiac imaging (e.g. CMR) has been used for the indication of congestive heart failure, cardiac tamponade, and acute myocardial infarction (Table 2) following a variety of imaging techniques. On the other hand, the elevation of troponin in critically ill patients is an indicator of silent myocardial inflammation in up to 28% of patients [26]. In athletes, identification of this form of disease that remains long after the resolution of typical COVID-19 symptoms is important before the resumption of training and competition.

**Fig. 2:**
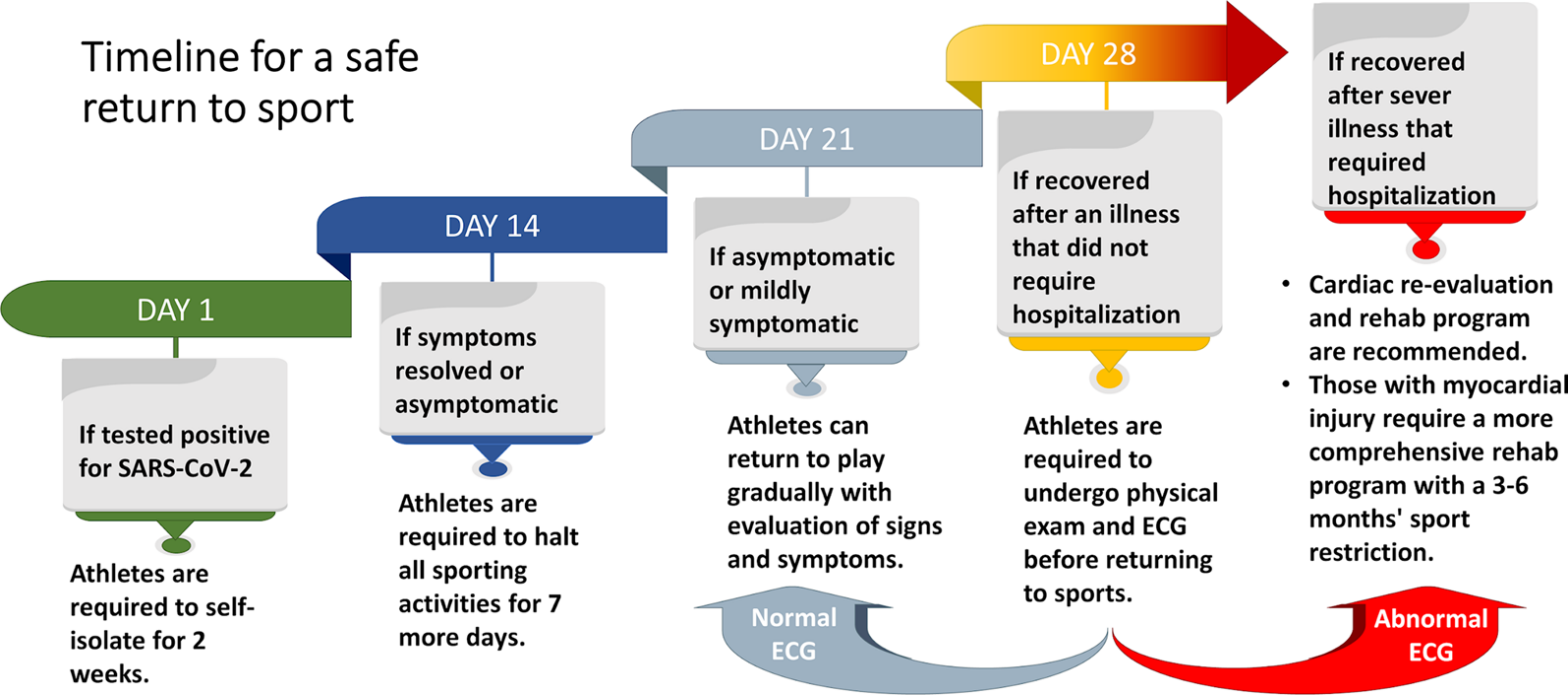
Recommended timeline for a safe return to sport following COVID-19 infection in athletes

Establishing a stepwise evaluation approach is critical with an emphasis on imaging techniques for proper diagnosis and risk assessment for a safe return to play. COVID-19 remains to be an obstacle to both athletes and sports organizations. Therefore, a comprehensive evaluation approach and a safe return to sport plan are warranted (Table 3; Fig. 2). Assessing athletes returning to sports following COVID-19 remains challenging in providing the best medical advice based on clinical evidence. Prior to returning to athletic activity, Drezner and colleagues recommended a written medical clearance that assesses various factors to develop an appropriate return to sports plan [27]. This may include evaluation of signs and symptoms, a 12-lead ECG with a physical examination, exercise test, echocardiogram, CMR, HOLTER monitoring, and cardiac biomarkers [28]. Further pulmonary follow-up and testing may also be required which include chest radiograph, spirometry, oxygen saturation during exercise, chest CT, and other pulmonary tests [27]. Hospitalized athletes with myocardial injury may undergo specific screening tests, a cardiac complication monitoring plan, and a more comprehensive rehabilitation program for a safe return to athletic activities (Table 3; Fig. 2)

**Table 3:** A summary of diagnostic approaches indicating heart injury and their cardiac interpretation

Test	Finding	Interpretation
Peri-epicardial LGE	Increase contrast uptake	● Active pericarditis causing fibrosis and/or edema Myocarditis causing regional damage
Myocardial LGE	Non-ischemic patterns	● Recovered or acute myocarditis
T1 and T2 MRI scans	Increase T1*	● Myocardial interstitial fibrosis
	Increase T2**	● Myocardial Edema
	Both increase	● Active inflammation
	T1 increased with normal T2	● Recovered with some myocardial fibrosis leftover
C-reactive protein (CRP)	Increase CRP	● Myocarditis or pericarditis
Natriuretic peptide (NP)	Elevation	● Myocardial injury

*T1: the time constant for regrowth of longitudinal magnetization

** T2: the time constant for decay/dephasing of transverse magnetization

A 2-week home isolation is advised following testing positive for SARS-CoV-2 regardless of presence of symptoms [26]. Seven days’ post-recovery and clearance of symptoms, a gradual return to sports activity is advised with continuous monitoring of any cardiac abnormalities that may appear [29]. Four weeks’ post-infection, athletes are advised to undergo cardiac re-evaluation if experiencing debilitating illness or reduced performance, with an immediate halt to all sporting activities [26]. If myocarditis is confirmed, a more intensive monitoring with 3–6 months’ sports restriction is required [28].

## **Summary**

Varied prevalence of cardiovascular complications were reported in all retrieved manuscripts including; myocarditis (0.4-15.4%), pericardial effusion (0.27-58%), pericarditis (0.06-2.2%), elevated troponin levels (0.9-6.9%) and myocardial edema. Early recognition of cardiac abnormality associated with myocarditis such as myocardial fibrosis, pericarditis, and edema are imperative to prevent sudden cardiac death in competitive athletes. For a safe return to athletic activities, COVID-19 remains to be an obstacle to both athletes and sports organizations. Therefore, a comprehensive evaluation approach and a safe return to sport plan are warranted.

## Data Availability

The datasets used and/or analyzed during the current study are available from the corresponding author on reasonable request.

## References

[1] Johns Hopkins Coronavirus Resource Center, COVID-19 Data in Motion (2022). In Internet https://coronavirus.jhu.edu/: Accessed January 30, 2022.

[2] Scudiero O, Lombardo B, Brancaccio M et al. Exercise, immune system, nutrition, respiratory and cardiovascular diseases during COVID-19: a complex combination. Int J Environ Res Public Health 2021;18. 10.3390/ijerph18030904 PMC7908487 33494244

[3] Camporota L, Chiumello D, Busana M (2021). Pathophysiology of COVID-19-associated acute respiratory distress syndrome. Lancet Respir Med.

[4] Kennedy FM, Sharma S (2020). COVID-19, the heart and returning to physical exercise. Occup Med (Lond).

[5] Barker-Davies RM, O’Sullivan O, Senaratne KPP (2020). The Stanford Hall consensus statement for post-COVID-19 rehabilitation. Br J Sports Med.

[6] Rajpal S, Tong MS, Borchers J (2021). Cardiovascular magnetic resonance findings in competitive athletes recovering from COVID-19 infection. JAMA Cardiol.

[7] Wilson MG, Hull JH, Rogers J (2020). Cardiorespiratory considerations for return-to-play in elite athletes after COVID-19 infection: a practical guide for sport and exercise medicine physicians. Br J Sports Med.

[8] Clark DE, Parikh A, Dendy JM, Diamond AB, George-Durrett K, Fish FA, Slaughter JC, Fitch W, Hughes SG, Soslow JH (2021). COVID-19 myocardial pathology evaluation in athletes with cardiac magnetic resonance (COMPETE CMR). Circulation.

[9] Brito D, Meester S, Yanamala N, Patel HB, Balcik BJ, Casaclang-Verzosa G, Seetharam K, Riveros D, Beto RJ, Balla S, Monseau AJ (2021). High prevalence of pericardial involvement in college student athletes recovering from COVID-19. Cardiovascular Imaging..

[10] Erickson JL, Poterucha JT, Gende A, McEleney M, Wencl CM, Castaneda M, Gran L, Luedke J, Collum J, Fischer KM, Jagim AR (2021). Use of electrocardiographic screening to clear athletes for return to sports following COVID-19 infection. Mayo Clin Proc: Innov Quality Outcomes.

[11] Hendrickson BS, Stephens RE, Chang JV, Amburn JM, Pierotti LL, Johnson JL, Hyden JC, Johnson JN, Philip RR (2021). Cardiovascular evaluation after COVID-19 in 137 collegiate athletes: results of an algorithm-guided screening. Circulation..

[12] Vago H, Szabo L, Dohy Z, Merkely B (2021). Cardiac magnetic resonance findings in patients recovered from COVID-19: initial experiences in elite athletes. Cardiovascular Imaging..

[13] Daniels CJ, Rajpal S, Greenshields JT, Rosenthal GL, Chung EH, Terrin M, Jeudy J, Mattson SE, Law IH, Borchers J, Kovacs R. Prevalence of clinical and subclinical myocarditis in competitive athletes with recent SARS-CoV-2 infection: Results from the big ten COVID-19 cardiac registry. JAMA Cardiol. 2021. 10.1001/jamacardio.2021.2065 PMC8160916 34042947

[14] Cavigli L, Frascaro F, Turchini F, Mochi N, Sarto P, Bianchi S, Parri A, Carraro N, Valente S, Focardi M, Cameli M. A prospective study on the consequences of SARS-CoV-2 infection on the heart of young adult competitive athletes: implications for a safe return-to-play. International journal of cardiology. 2021. 10.1016/j.ijcard.2021.05.042 PMC8166156 34082008

[15] Martinez MW, Tucker AM, Bloom OJ, Green G, DiFiori JP, Solomon G, Phelan D, Kim JH, Meeuwisse W, Sills AK, Rowe D. Prevalence of inflammatory heart disease among professional athletes with prior COVID-19 infection who received systematic return-to-play cardiac screening. JAMA cardiology. 2021. 10.1001/jamacardio.2021.0565 PMC7934073 33662103

[16] Małek ŁA, Marczak M, Miłosz-Wieczorek B, Konopka M, Braksator W, Drygas W, Krzywański J (2021). Cardiac involvement in consecutive elite athletes recovered from Covid‐19: A magnetic resonance study. Journal of Magnetic Resonance Imaging.

[17] Cavarretta E, D’Angeli I, Giammarinaro M, Gervasi S, Fanchini M, Causarano A, Costa V, Manara M, Terribili N, Sciarra L, CalÒ L. Cardiovascular effects of COVID-19 lockdown in professional Football players. Panminerva Medica. 2021. 10.23736/S0031-0808.21.04340-8 33565761

[18] Mascia G, Pescetelli F, Baldari A, Gatto P, Seitun S, Sartori P, Pieroni M, Calò L, Della Bona R, Porto I (2021). Interpretation of elevated high-sensitivity cardiac troponin I in elite soccer players previously infected by severe acute respiratory syndrome coronavirus 2. Int J Cardiol.

[19] Moulson N, Petek BJ, Drezner JA, Harmon KG, Kliethermes SA, Patel MR, Baggish AL. SARS-CoV-2 cardiac involvement in young competitive athletes. Circulation. 2021. 10.1161/CIRCULATIONAHA.121.054824 PMC8300154 33866822

[20] Gervasi SF, Pengue L, Damato L, Monti R, Pradella S, Pirronti T, Bartoloni A, Epifani F, Saggese A, Cuccaro F, Bianco M (2021). Is extensive cardiopulmonary screening useful in athletes with previous asymptomatic or mild SARS-CoV-2 infection?. British Journal of Sports Medicine.

[21] Starekova J, Bluemke DA, Bradham WS, Eckhardt LL, Grist TM, Kusmirek JE, Purtell CS, Schiebler ML, Reeder SB. Evaluation for myocarditis in competitive student athletes recovering from coronavirus disease 2019 with cardiac magnetic resonance imaging. JAMA cardiology. 2021. 10.1001/jamacardio.2020.7444 PMC7809616 33443537

[22] Murad MH, Sultan S, Haffar S (2018). Methodological quality and synthesis of case series and case reports. BMJ Evid Based Med.

[23] Shi S, Qin M, Shen B (2020). Association of Cardiac Injury With Mortality in Hospitalized Patients With COVID-19 in Wuhan, China. JAMA Cardiol.

[24] Puntmann VO, Carerj ML, Wieters I (2020). Outcomes of cardiovascular magnetic resonance imaging in patients recently recovered from coronavirus disease 2019 (COVID-19). JAMA Cardiol.

[25] Huang L, Zhao P, Tang D (2020). Cardiac involvement in patients recovered from COVID-2019 identified using magnetic resonance imaging. JACC Cardiovasc Imaging.

[26] Baggish A, Drezner JA, Kim J (2020). Resurgence of sport in the wake of COVID-19: cardiac considerations in competitive athletes. Br J Sports Med.

[27] Drezner JA, Heinz WM, Asif IM (2020). Cardiopulmonary Considerations for High School Student-Athletes During the COVID-19 Pandemic: NFHS-AMSSM Guidance Statement. Sports Health.

[28] Verwoert GC, de Vries ST, Bijsterveld N (2020). Return to sports after COVID-19: a position paper from the Dutch Sports Cardiology Section of the Netherlands Society of Cardiology. Neth Heart J.

[29] Bhatia RT, Marwaha S, Malhotra A (2020). Exercise in the Severe Acute Respiratory Syndrome Coronavirus-2 (SARS-CoV-2) era: A Question and Answer session with the experts Endorsed by the section of Sports Cardiology & Exercise of the European Association of Preventive Cardiology (EAPC). Eur J Prev Cardiol.

